# Ex vivo rescue of recombinant very virulent IBDV using a RNA polymerase II driven system and primary chicken bursal cells

**DOI:** 10.1038/s41598-020-70095-x

**Published:** 2020-08-06

**Authors:** Liliana L. Cubas-Gaona, Romane Trombetta, Céline Courtillon, Kai Li, Xiaole Qi, Xiaomei Wang, Sofiane Lotmani, Alassane Keita, Michel Amelot, Nicolas Eterradossi, Sébastien Mathieu Soubies

**Affiliations:** 1 French Agency for Food Safety (ANSES), Maisons-Alfort, France; 2 Division of Avian Infectious Diseases, State Key Laboratory of Veterinary Biotechnology, Harbin Veterinary Research Institute, Chinese Academy of Agricultural Sciences, Harbin, 150069 P.R. China

**Keywords:** Virology, Viral genetics, Microbiology techniques

## Abstract

Infectious Bursal Disease Virus (IBDV), a member of the *Birnaviridae* family, causes an immunosuppressive disease in young chickens. Although several reverse genetics systems are available for IBDV, the isolation of most field-derived strains, such as very virulent IBDV (vvIBDV) and their subsequent rescue, has remained challenging due to the lack of replication of those viruses in vitro. Such rescue required either the inoculation of animals, embryonated eggs, or the introduction of mutations in the capsid protein (VP2) hypervariable region (HVR) to adapt the virus to cell culture, the latter option concomitantly altering its virulence in vivo. We describe an improved ex vivo IBDV rescue system based on the transfection of an avian cell line with RNA polymerase II-based expression vectors, combined with replication on primary chicken bursal cells, the main cell type targeted in vivo of IBDV. We validated this system by rescuing to high titers two recombinant IBDV strains: a cell-culture adapted attenuated strain and a vvIBDV. Sequencing of VP2 HVR confirmed the absence of unwanted mutations that may alter the biological properties of the recombinant viruses. Therefore, this approach is efficient, economical, time-saving, reduces animal suffering and can be used to rescue other non-cell culture adapted IBDV strains.

## Introduction

Infectious Bursal Disease Virus (IBDV) is responsible for an immunosuppressive and sometimes fatal disease in chickens also called Gumboro disease^1^, that causes large economic losses in the poultry sector worldwide^2^. IBDV belongs to the *Birnaviridae* family and is the only species of the genus *Avibirnavirus*. The naked viral particle, about 60 nm in diameter, contains two segments of double-stranded RNA (dsRNA) of 3.2 and 2.8 kilo base pairs called segment A and B, respectively^3^. Segment A harbors two partially overlapping open reading frames (ORFs). The first one codes for VP5, a non-structural protein dispensable for virus replication which has been suggested to possess a crucial role in the cell-to-cell transmission of the virus^4^. The second one encodes a polyprotein, which is self-processed upon co-translational cleavage, yielding the capsid precursor protein pVP2, the multifunctional nucleoprotein VP3 and the viral protease VP4. Further processing and cleavage events by VP4 and VP2 itself yield mature VP2. The capsid protein can be divided into three domains named base (B), shell (S) and projection (P)^5^, the P domain contains the hypervariable region (HVR), which spans from residues 206 to 350 and elicits neutralizing antibodies^6,7^. Segment B contains only one ORF which encodes VP1, the RNA-dependent RNA polymerase (RdRp)^8^.

Two serotypes have been described for IBDV based on cross-neutralization assays^9^. Serotype 1 viruses are further subdivided into classical, very virulent (vv) and subclinical antigenic variants strains based on pathogenicity studies. Serotype 1 viruses infect and destroy immature B lymphocytes contained in the Bursa of Fabricius (BF) of young chickens. This destruction, that can be associated to transient or permanent histological damages of the BF, leads to an immunosuppression^10^. In contrast, serotype 2 strains are non-pathogenic and neither cause disease in poultry nor protect against serotype 1 infections^1^.

Various cell lines have been used to study and propagate IBDV strains. They include primary chicken embryo fibroblasts (CEF), cell lines originating from chicken (DF-1, DT40 or the B-lymphoblastoid cell lines LSCC-BK3 and LSCC-CU10)^1^, quail-derived cells (QM7, QM5 or QT35) and mammalian cell lines such as African Green Monkey Vero or human HeLa cells^11,12^. Although vvIBDV replication has been reported in DT40^13^, LSCC-BK3 and LSCC-CU10^14^, pathogenic field strains such as vvIBDV do not replicate easily in those in vitro systems. Adaptation to cell culture is possible and is associated to mutations in positions 253, 279, 284 and 330 in VP2 HVR. Nevertheless, these mutations generally result in viral attenuation in chickens^15–17^.

The development of reverse genetic systems enabled breakthroughs in IBDV research. Therewith, the manipulation of IBDV genome and study of viral proteins have become possible. The first IBDV rescue system, reported at the end of 90 s, was based on in vitro transcription by T7 RNA polymerase of full-length cDNA clones of segments A and B, both with cap analogs^18^, but this approach was expensive and complicated to perform. Later, to rescue vvIBDV, the direct transfection of cells with full-length cDNA cloned downstream of T7 promoter and followed by hepatitis delta virus (HDV) ribozyme -to ensure the generation of correct 3′ ends- and T7 terminator was described^19,20^. However, the T7 polymerase needs to be provided *in trans*, for example by a recombinant fowlpoxvirus; the rescued recombinant IBDV must then be separated from this helper virus. In an attempt to develop an easier and better method to rescue IBDV, Qi et al.^21^ used a reverse genetics platform based on RNA polymerase II (pol II). They produced recombinant eukaryotic expression vectors containing the full-length cDNA sequences of segment A and B of IBDV Gt strain, flanked by hammerhead (HH) and HDV ribozymes and downstream of cytomegalovirus (CMV) enhancer and a beta chicken actin promoter. A simplified view of this approach was described in which HH ribozyme was replaced by fusing the 5′ terminal sequence of the cDNA of each segment to the intermediate early CMV promoter, while the authors kept the ribozyme sequence at the 3′ ends. This strategy resulted in a high yield of infectious particles^22^. Recently, a new approach for the recovery of IBDV was described, in which the trans-supplementation of the viral VP1 and VP3 proteins was required and sufficient to rescue infectious virus from RNA polymerase I (pol I) transcribed IBDV genomic RNA^23^. This strategy was further improved through the design of a dual promoter plasmid system, which includes, upstream of the pol I promoter ensuring genomic RNA synthesis , a pol II promoter that results in the expression of viral proteins^24^.

Typically, rescue of recombinants strains adapted to cell culture is performed by transfecting cell lines such as QM7^4^, Vero^25^ or primary CEF^26^. Rescue of non-cell culture adapted recombinant viruses has been achieved by the transfer of supernatants and/or lysates from transfected cell lines to specific pathogen-free (SPF) chickens embryonated eggs—via the chorioallantoic membrane route^1,20^- or SPF chickens, which are inoculated by different routes such as intrabursal^27^, intramuscular routes^28^ or by eye-drop^15^. Besides the impact on animal welfare, the use of numerous chickens or embryonated eggs to rescue non-cell culture adapted viruses makes this process laborious and time-consuming. In consequence, an efficient, economical and improved method to easily rescue viruses such as vvIBDV is required.

We recently showed that primary bursal cells stimulated with Phorbol Myristate Acetate (PMA) could be used to propagate to high titers not only cell culture adapted virus exhibiting bursal tropism, but also vvIBDV or antigenic variant without any adaptation^29^. Based on this advance, the present study describes an efficient ex vivo system for IBDV rescue. This system is based on pol II-driven expression of viral RNA (vRNA) segments in transfected DF-1 cells, followed by transfer of this cellular material onto chicken primary B cells. Using this system, we rescued two recombinant IBDV strains: a cell-culture adapted, attenuated strain, Cu-1, and a very virulent IBDV, not adapted to cell culture, rvv. Sequencing of the VP2 HVR of the rescued viruses revealed the absence of mutation in this region.

## Results

### Specific VP3 expression after co-transfection with either prACu-1 and prBCu-1 or prAvv and prBvv in DF-1 cells

We firstly attempted direct transfection of a plasmid that expressed green fluorescent protein (GFP) into primary bursal cells, by either lipofection or electroporation. Both strategies failed and were quickly abandoned (data not shown). Instead, DF-1 cells, which have been previously used for IBDV rescue^23,30^, were chosen as an intermediate host to rescue the recombinant viruses Cu-1 (rCu-1) and vv (rvv).

**Figure 1:**
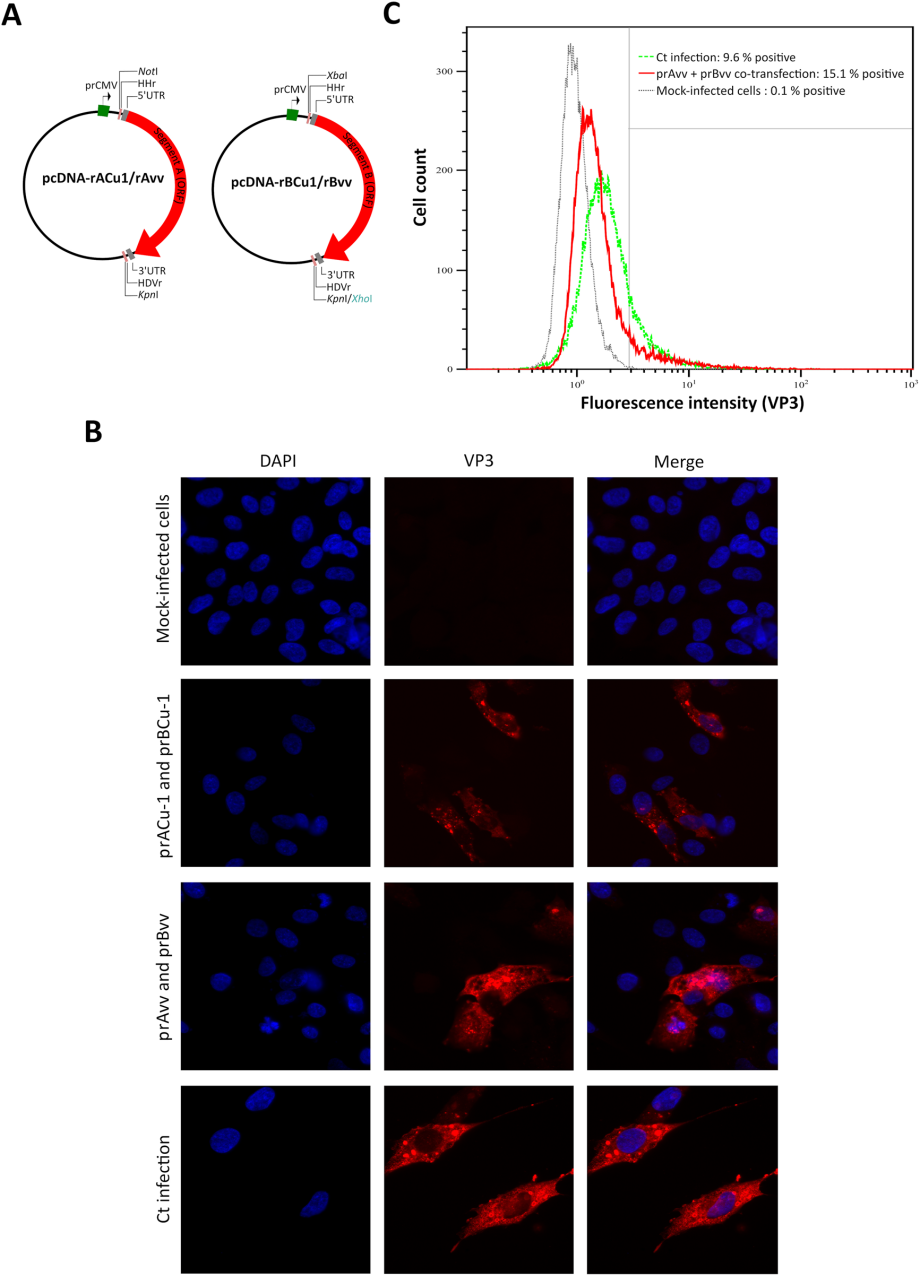
DF-1 cells co-transfected with either prACu-1 and prBCu-1 or prAvv and prBvv specifically express VP3 viral protein. (**A**) Schematic representation of expression vectors prACu-1 or prAvv and prBCu-1 or prBvv, which express segments A and B of strains rCu-1 and rvv, respectively. *prCMV* cytomegalovirus promoter, *HHr* Hammerhead ribozyme, *HDVr* hepatitis delta virus ribozyme. Restriction sites for the enzymes *Not*I, *Kpn*I, *Xba*I and *Kpn*I (for expression vector prBCu-1)/*Xho*I (for expression vector prBvv) were used for the construction of recombinant vectors. (**B**,**C**) DF-1 cells were either mock-infected, infected with viral strain Ct (multiplicity of infection of 0.01) or co-transfected with either prACu-1 and prBCu-1 or prAvv and prBvv and analyzed by immunofluorescent test (**B**) and, for cells co-transfected with prAvv and prBvv, by Flow Cytometry (**C**). DAPI staining appears in blue and VP3 staining appears in red.

In a first step, to check if viral protein expression took place upon transfection, DF-1 cells were co-transfected with the recombinant expression vectors, either prACu-1 and prBCu-1, or prAvv and prBvv (Fig. 1A), designed to allow rCu-1 and rvv recovery, respectively. Analysis by immunofluorescent tests showed the specific accumulation of VP3 in cells co-transfected with prACu-1 and prBCu-1 (Fig. 1B, second row) and in cells co-transfected with prAvv and prBvv (Fig. 1B, third row). VP3 cytoplasmic accumulation pattern was similar to what was observed after infection with the cell culture adapted viral strain Ct^31^, used as positive control (Fig. 1B, forth row). In addition, similar percentages of VP3-positive cells were observed upon co-transfection with either prAvv and prBvv (15.8% positive cells) or prACu-1 and prBCu-1 (21.2% positive cells). Since vvIBDV replication is not possible in cell culture without adaptation, making rescue of rvv more challenging, we additionally quantified the proportion of transfected cells upon co-transfection with prAvv and prBvv in comparison with infected cells by flow cytometry. This approach revealed around 15% VP3-positive cells upon co-transfection with prAvv and prBvv, a percentage similar to the one obtained by immunofluorescent test, and about 9% positive cells upon Ct infection (Fig. 1C). These results showed that transfected DF-1 cells expressed VP3, a prerequisite for the production of infectious viral particles.

### Transfer of transfected DF-1 onto chicken primary B cells results in the production of high titer stocks of rescued viruses

**Figure 2:**
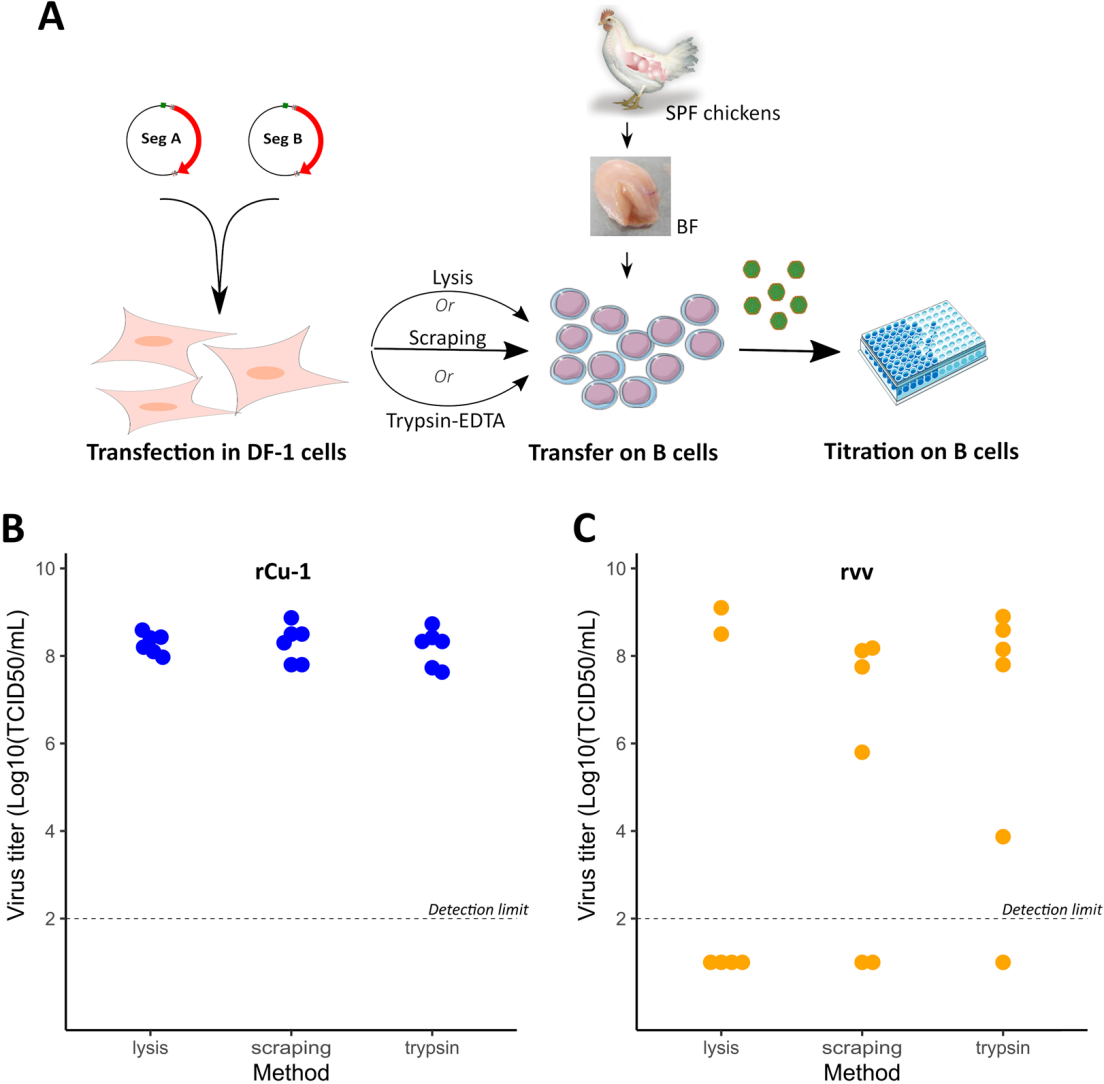
Transfer of co-transfected DF-1 onto primary B cells results in the production of molecular clones rCu-1 and rvv. (**A**) Complete schematic representation of IBDV rescue. (**B**) DF-1 cells were co-transfected with plasmids that express segment A and B for each molecular clone. After 72 h, cellular material was (i) lysed by three freeze–thaw cycles, (ii) mechanically detached or (iii) enzymatically detached using trypsin–EDTA and transferred onto B cells (10^7^ cells/ml) seeded in 75 cm^2^ flask. Cultures were harvested 48 h post-transfer and supernatants were titrated. Results are from three independent experiments each with two biological replicates.

Since bursal cells are highly permissive to IBDV infection, we hypothesized that transfer of supernatant and cellular material from transfected DF-1 cultures might be sufficient to infect primary B cells. For this purpose, three different methods of transfer were tested to optimize the efficiency of rCu-1 and rvv rescues (Fig. 2A). Seventy-two hours post-transfection with either prACu-1 and prBCu-1 or prAvv and prBvv, DF-1 cultures were transferred onto B cells after being either (i) lysed by three freeze–thaw cycles, (ii) mechanically detached using a cell scraper (hereafter referred as “scraping”) or (iii) enzymatically detached using trypsin–EDTA (hereafter referred as “enzymatic method”). The B cell cultures were harvested 48 h post-transfer and supernatants were titrated. Three independent rescue experiments were carried out, each one with two replicates per condition. Recovery of rCu-1 was successful in all our attempts, with titers ranging from 10^8.0^ to 10^8.9^ mean tissue infectious doses per ml (TCID_50_/mL, Fig. 2B) irrespectively of the method used. In contrast, the success rate of rvv recovery varied depending on the transfer method: 33% for lysis, 66% for scraping and 83% for the enzymatic method were reached. Successful recovery of rvv was associated with mean viral titers of 10^8.8^ TCID_50_/mL, 10^7.4^ TCID_50_/ml and 10^7.5^ TCID_50_/ml for lysis, scraping and enzymatic method, respectively (Fig. 2C).

As scraping and enzymatic methods showed higher success rates than lysis method for the recovery of rvv, with at least one viral stock per experiment, those transfer methods, and not the lysis method, were kept for further experiments.

### No modification in VP2 hypervariable region is detected after rCu-1 and rvv rescue

**Figure 3:**
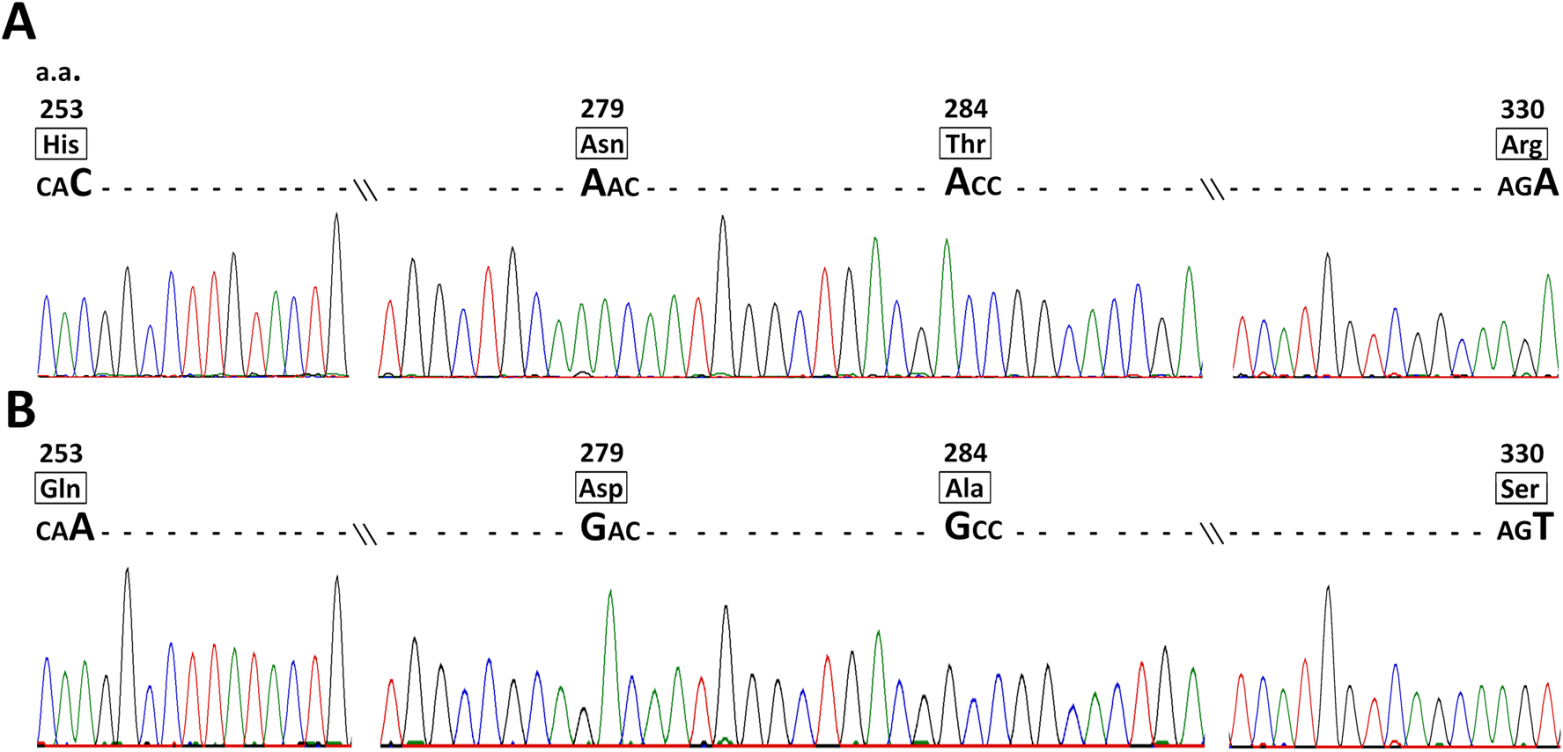
VP2 hypervariable region remains unmodified after rCu-1 and rvv rescue. VP2 HVR of the recombinant viruses rCu-1 (**A**) and rvv (**B**) were recovered by RT-PCR and sequenced. Sequencing results were compared with the consensus sequence of each strain. Both schemes show in black squares the amino acidic signature for each recombinant virus as well as the corresponding codon for each amino acid. The bigger letter represents the nucleotide change for each codon between both strains. A dashed line replaced the remaining nucleotide sequence.

Since VP2 HVR sequence variations are associated to adaptation to cell culture, this region was sequenced to exclude any modification of cell tropism as a result of replication on B cells and to confirm the identity of rescued viruses. Sequencing of VP2 HVR was performed on three stocks of both rCu-1 and rvv recovered from independent rescue experiments using the enzymatic transfer method (Fig. 3). The obtained sequences confirmed the identity of each rescued viruses. Importantly, no mutation was observed in VP2 HVR: three out of three rCu-1 stocks exhibited a His253-Asn279-Thr284-Arg330 signature while all sequenced rvv stocks (3/3) presented a Gln253-Asp279-Ala284-Ser330 signature. No significantly high subpeak was detected on the chromatograms for these positions, arguing against any modification of VP2 HVR, potentially modifying virus tropism, immediately after rescue on bursal cells.

## Discussion

Since its first application to recover poliovirus in 1981^32^, reverse genetics has provided critical insights into the replication and pathogenicity of animal RNA virus and has helped to improve vaccine development^33^.

Very virulent IBDV first appeared at the end of the eighties before spreading worldwide and causing up to 70% mortality in the field^34,35^. Several approaches have been used to rescue vvIBDV in order to understand the molecular basis of the increased pathogenicity of this pathotype. However, this understanding is limited because the isolation of field-derived IBDV such as vvIBDV and their subsequent rescue in cell lines only may result in adaptation to cell culture, which is correlated with attenuation in chickens. To avoid this bias, most of these approaches used several chickens as a final host in the rescue process. The first successful attempt to rescue a vvIBDV strain from cloned cDNA using B cells, through previous transfection in QM5 cells, was described by Boot et al.^20^. However, this system depended on recombinant fowlpox virus containing the T7 polymerase gene poxvirus. Other approaches used in vitro synthesis of RNA, transfection on CEF and rescue in chickens^36^. Qi et al.^21^ developed an improved method for the rescue of an attenuated IBDV strain employing RNA pol II system to drive RNA synthesis, in combination with HH and HDV ribozymes. Taking advantage of this pol II-system and combining it with the use of primary bursal B cells, the present study describes the rescues of two recombinant strains: rCu-1, a strain attenuated and adapted to cell culture, and rvv, which is a very virulent IBDV not adapted to cell culture. This was possible by transferring the supernatant and cellular material from transfected DF-1 cells (permissive for rCu-1 propagation, but not for rvv) to primary B cells (permissive for both viruses). This system enabled to improve the efficiency and reduce the costs of recombinant vvIBDV recovery. This strategy reduces the use of chickens because for this purpose, one bursa yields enough cells to rescue several stocks while approaches using chicken inoculation required several animals to rescue one single virus. As primary bursal B cells are permissive for both IBDV strains, our first attempts used exclusively B cells for IBDV recue. However, we found transfection of B cells to be inefficient (data not shown). Transfecting sensitive and non-adherent primary cells such as bursal B cells with high efficiency while preserving their viability remains a challenge. Although different vvIBDV strains have been shown to replicate in LSCC-BK3, LSCC-CU10 and DT40 cell lines^14,37,38^, we decided not to use them for three main reasons. (i) No study about of their use in the IBDV rescue has been reported, (ii) slow viral replication associated with the appearance of mutations in VP2 HVR^38^ in comparison with our results obtained on B cells^29^ and (iii) the risk of contaminating rescued IBDV stocks with avian leucosis virus (ALV) since those cell lines were obtained from ALV-induced tumors. Instead, DF-1 cells appeared to be a good alternative for IBDV rescue, because they have been used for this approach, including to rescue vvIBDV^27^. Additionally, they are easier to cultivate compared with primary cells.

Although both electroporation and lipofection were initially tested to transfect DF-1 cells (data not shown), we chosed lipofection as a transfection method since this technique, at least in ours hands, requires lower amounts of plasmids and better preserves cell viability. Preliminary lipofection tests with various quantities of plasmids were carried out: no measurable difference was observed in protein expression levels measured at 72 h post-transfection (data not shown), a standard time in transfection experiments^23^. We thus used the amount of plasmids recommended by the manufacturer in subsequent experiments. Our rescue vectors, as well as those described by Qi et al.^21^, rely on the cleavage of the transcribed RNA by the HH and HDV ribozymes. This cleavage is supposed to result in the production of positive-stranded genomic vRNAs with precisely trimmed extremities: these vRNAs seem to be sufficient to rescue IBDV. This model appears to be in contradiction with recent data showing that both VP3 and VP1 proteins are necessary, in addition to viral genomic RNAs for the production of infectious particles after transfection^20^. Those data are moreover consistent with another work showing that VP1 and VP3 associate with the genomic vRNA to form transcriptionally-competent ribonucleoprotein (RNP) complexes^23,39^. This apparent paradox with our data may be solved by taking into account a suboptimal efficiency of ribozyme-mediated cleavage of the transcribed RNA. For instance, the kinetics of HH ribozyme cleavage was shown to be dependent on its subcellular localization^40,41^. This phenomenon could leave some uncleaved, capped and polyadenylated “viral-like” RNA: those could be translated, resulting in at least low amounts of viral proteins, including VP1 and VP3. Those proteins could then associate with correctly cleaved vRNA segments: the resulting RNP complexes would be sufficient to initiate viral replication. This hypothesis is in agreement with (i) data from the first IBDV rescue system in which translation was possible upon transfection of capped transcribed RNA generated in vitro^18^. (ii) More recent data showing that capped but not uncapped IBDV RNA transcripts are sufficient to rescue the virus^23^ and (iii) our data showing expression of VP3 in transfected DF-1 cells,VP3 expression was indeed also observed when DF-1 cells were transfected with pcDNA-rAvv alone (data not shown).

Although we observed expression of VP3 protein, we anticipated that rescue of rCu-1 would be easier than rvv. As long as a few infectious particles of a cell-culture adapted IBDV such as rCu-1 are produced upon transfection, they can further replicate in DF-1 cells, increasing the infectious titer in the well^4,25^. On the contrary, in the case of rvv, a first artificial replication cycle may take place in DF-1 cells after transfection of the rescue vectors. Nevertheless, the few resulting viral particles, harboring the VP2 Gln253, Asp279, Ala284 and Ser330 signature, will not be able to further replicate in DF-1 cells: in this case, the recovery of rvv crucially depends on the yield of infectious particles after transfection. Preliminary titration experiments performed after transfection and prior to transfer onto B cells failed to detect (case of rvv) or to quantify (case of rCu-1) infectivity (data not shown). Permissive cells such as B cells were thus required after transfection, in particular for rvv rescue, in order to obtain high-titer viral stocks.

In order to optimize the efficiency of our IBDV rescue protocol, we assayed three different methods, lysis, scraping and enzymatic, to transfer cellular material from transfected DF-1 onto primary B cells. Cell lysis by repeated freeze–thaw cycles is a classical approach in virology that is supposed to help releasing intracellular viral particles; it has been used in several IBDV rescue protocols^15,21,23^. Alternatively, the aim of the scraping and enzymatic methods is to establish co-cultures. Such co-culture approaches between one cell type efficiently transfected and one cell type highly permissive to viral replication have been used to establish a high yield of progeny virus in several reverse genetics systems. For example, the rescue of several influenza A viruses has been achieved using a co-culture of Vero and CHOK1 cells or 293 T and MDCK cells^42,43^. The transfection of both mammalian and mosquito cells with vRNA transcripts has been used to improve the rescue of dengue virus^44^. With respect to rCu-1 rescue, the transfer method used irrespectively yielded productive infectious particles with reproducible and high titers. This result was expected since rCu-1 is a cell culture adapted strain capable to replicate in DF-1 and B cells. However, for rvv rescue, different tendencies depending on the transfer method were observed, although the overall number of experiments did not allow us to perform statistical analyses. In particular, the lysis method resulted in less successful rescues. This may be due to the repeated freeze–thaw cycles, which may physically alter infectious particles and reduce global infectivity.

Alternatively, co-culture methods (scraping and enzymatic detachment) seemed to improve the success rate of vvIBDV rescue. In this case, the preserved viability of DF-1 cells after transfer may allow to maintain production of viral proteins for a longer period. Between these two methods, the enzymatic method gave more successful recoveries. One reason could be that after enzymatic detachment, DF-1 cells are individualized and are able to form a well-established monolayer, promoting close contact with B cells and improving viral transmission. In our laboratory, we chose to use the enzymatic method, which is simpler from a practical point of view. Readers may want to test both co-culture methods and choose which one works better in their settings.

An ideal rescue system for IBDV should not favor the appearance of unwanted mutations, in particular in VP2 HVR. Four amino acid substitutions have been associated with tissue culture adaptation and attenuation in chickens of viruses such as rvv: Gln253His, Asp279Asn, Ala284Thr and Ser330Arg^16,45,46^. For that reason, the VP2 HVR of each rescued IBDV was checked for conformity with the expected construction by nucleotide sequencing. In particular, we did not find any change in the VP2 HVR rvv, which shows that one passage on B cells does not result in mutations in this region.

The rescue of rCu-1, a strain adapted to cell culture, could have been performed using DF-1 cells only. We nonetheless used B cells after DF-1 cells transfection to benefit from the high replicative capacity of IBDV in this cell type and obtain high-titer stocks. Serial passage in chickens of an attenuated vaccine strain has been associated with reversions in VP2 positions 253 and/or 284^17,47^, additionally, vaccine-related viruses harboring those changes and associated to severe bursal damages have been detected in the field^47^. It was thus important to check if replication of rCu-1 in B cells during the rescue protocol would result in the appearance of those changes. Again, no mutation was found in rCu-1 VP2 HVR, illustrating the versatility of our system to rescue various types of recombinant IBDV strains without unwanted mutations. It will be of interest to study the evolution of IBDV strains with different properties such as rCu-1 and rvv upon serial passage on B cells.

Several strategies may be of interest to further optimize this rescue protocol. First, transfection parameters such as the quantity of plasmid used or the timing for transfer may be adapted to each laboratory settings in order to maximize viral material expression. Second, the extremely low infectivity levels observed immediately after transfection may be increased. Plasmid DNA transfection has been shown to inhibit virus infection^48^. This may be explained by sensing of plasmid DNA by the cGAS/STING system, followed by the induction and secretion of type I interferons^49^ (IFNs), resulting in an antiviral state. Thus, the transfection of cells genetically deficient in IFN response or the use of chemical inhibitors either upstream of IFN induction (such as the TBK1/ IKK*ε* inhibitor BX-795^50^ or downstream of IFN signalling (such as the JAK1/2 inhibitor Ruxolitinib^51^ may help to optimize the viral rescue.

In conclusion, this improved system to IBDV rescue offers two main advantages. First, the combination, for the first time, of the polymerase II system with IBDV replication in primary B cells makes possible the rescue of recombinant IBDV strains, irrespective of their adaptation to cell culture, to high titers. Second, this system combines a reduced use of animals for IBDV research with an increased capacity to generate recombinant viruses in one round. We hope this system will help to provide new insight into IBDV biology.

## Material and methods

### Viruses and plasmids

rCu-1 is a cell-culture adapted attenuated strain whose origin was reported previously^52^. The recombinant strain rvv is a very virulent IBDV whose sequence is a consensus from the coding regions of the typical vvIBDV strains D6948, HK46, BD3 and UK661^36^.

The principle of the construction of the full-length IBDV cDNA clones as well as HH and HDV ribozyme sequences were previously described by Qi et al.^21^. Briefly, to generate both molecular clones, rCu-1 and rvv, unique restriction enzyme sites as well as HH and HDV ribozyme sequences were introduced by PCR into segment A and B of each IBDV strain. The PCR products were digested and ligated into pcDNA3.1 (−) expression vector (Invitrogen) to obtain the recombinant expression vectors prACu-1, prBCu-1, prAvv and prBvv (Fig. 1A). The four expression vectors were entirely sequenced to confirm the absence of unwanted mutations.

### DF-1 cells

DF-1 cell line (chicken embryonic fibroblast; ATCC CRL-12203) was grown in Dubelcco’s modified minimal essential medium (DMEM) (reference 61965-023, Thermo Fisher) supplemented with 10% fetal bovine serum (FBS), penicillin (200 IU/ml), streptomycin (0.2 mg/ml), fungizone (2 µg/ml) and maintained at 39 °C in a humidified 5% CO_2_ incubator.

### Transfection of DF-1 cells

DF-1 cells were grown in six-well plates to 70% confluence and co-transfected with 1.25 µg of each plasmid construct expressing segments A or B for each molecular clone using Lipofectamine 2000 reagent (reference 11668-027, Thermo Fisher) at a 1:2 ratio ( µg DNA : µl reagent), according to the manufacturer’s instructions. Briefly, the DNA-reagent mix was prepared in 250 µl Opti-MEM (reference 51985-026, Thermo Fisher) and incubated at room temperature for 15 min. The culture medium was replaced by fresh supplemented DMEM, the mixture was added dropwise into the dish and incubated at 39 °C during 72 h.

### Primary chicken bursal cell isolation

The raising of chickens, their euthanasia and the sampling of Bursae of Fabricius were conducted in an approved laboratory for animal experiments (n°C-22-745-1), in agreement with EU directive number 2010/63/UE and were approved by the ANSES Ploufragan laboratory local committee for animal welfare. Then, the Bursae of Fabricius were collected aseptically from 4 to 10 week-old SPF White Leghorns chickens (ANSES, Ploufragan, France) and processed as described in Soubies et al.^29^. Briefly, the cloacal bursae were dissociated with a tissue grinder homogenizer kit (reference CD1-1KT, Sigma-Aldrich), collected in sterile PBS and subjected to density gradient centrifugation. Bursal cells, in the opaque interphase, were recovered, washed once in PBS and maintained in lymphocyte culture medium (see composition below) at 40 °C in a humidified 5% CO_2_ incubator.

Lymphocyte culture medium was prepared using Iscove’s modified Dulbecco’s Medium (IMDM) with L-glutamine and HEPES (reference 21980-032, Gibco, Thermo Fisher) supplemented with 8% FBS, 2% SPF chicken serum (ANSES, Ploufragan, France), 1X insulin transferrin selenium (reference 41400-045, Gibco, Thermo Fisher), 50 μM beta-mercaptoethanol, 1 µg/ml PMA (reference tlrl-pma, Invivogen), penicillin (200 IU/ml), streptomycin (0.2 mg/ml) and fungizone (2 μg/ml). PMA was prepared as previously described by Soubies et al.^29^. To estimate bursal cell viability after isolation and adjust cell concentration, bursal cells were mixed with PBS containing 0.1% (m/v) erythrosin B (reference 200964, Sigma-Aldrich), a red dye which stains the dead cells, and counted in a Newbauer chamber.

### Virus titration by immunocytochemistry (ICC)

Ten-fold serial dilutions of supernatants from B cells were performed in IMDM and distributed into 96-well U bottom plates (50 µl/well, eight replicates per viral sample). Freshly prepared B cells in lymphocyte culture medium (10^6^ cells in 150 µl/well) were added in each well and incubated at 40 °C for 48 h. After this time, cells were washed with PBS and fixed with ethanol and acetone solution (1:1 ratio) at − 20 °C for at least 30 min. The fixation solution was removed and the plates were air-dried under a chemical hood and either processed immediately or stored at − 20 °C until further processing. Plates were subjected to ICC as described in Soubies et al.^29^ Titers were calculated using Reed and Munch formula and expressed as TCID_50_/ml.

### Flow cytometry

DF-1 cells were co-transfected as described above or infected with strain Ct at a multiplicity of infection (MOI) of 0.01. After 24 h, the cells were detached with trypsin–EDTA and washed with PBS 5% FBS, followed by fixation with Fixation buffer, washed twice with Permeabilization buffer (reference 88-8824, ThermoFisher) and incubation with mouse anti-VP3 monoclonal antibody (clone IBDV9, reference 3BD5, Hytest). After three washes with permeabilization buffer, cells were incubated with a goat anti-mouse IgG2a Alexa Fluor 546 (reference A21133, ThermoFisher), washed again three times with permeabilization buffer and analyzed with a FC500 MPL flow cytometer (Beckman Coulter). The fixation step and antibody incubations were carried out during 30 min at room temperature in the dark.

### Immunofluorescence

DF-1 cells seeded onto glass coverslip in 24-well plates were infected with Ct viral stock at a MOI of 0.01 or co-transfected as described above with 0.4 µg of each plasmid construct expressing segments A or B. After 24 h, coverslips were washed with PBS, fixed with ethanol:acetone solution (1:1 ratio) for 20 min at − 20 °C and dried for 15 min under chemical hood. Coverslips were blocked for 30 min using PBS containing 5% FBS. Later, samples were incubated for 1 h with mouse anti-VP3 monoclonal antibody (clone IBDV9, reference 3BD5, Hytest) diluted in PBS 5% FBS. Coverslips were washed three times with PBS and incubated for 30 min with a goat anti-mouse IgG2a Alexa Fluor 546 (reference A21133, ThermoFisher) and Hoechst 33342 ( reference 14533, Sigma-Aldrich) diluted in PBS 5% FBS. All incubations were performed at room temperature. Finally, coverslips were dried and mounted with ProLong diamond antifade mountant (reference P36965, Thermofisher). Samples were visualized with an Olympus BX41 inverted fluorescence microscope.

### RNA extraction, partial amplification and sequencing

Viral RNA was isolated using the QIAamp viral RNA mini kit (reference 52904, Qiagen). Segment A and B were partially reverse transcribed into cDNA by Maxima H minus Reverse Transcriptase (reference EP0752, ThermoFisher) according to the manufacturer’s protocol, using chimeric primers described previously^53^. The reaction was incubated at 50 °C for 30 min and heated at 85 °C for 5 min to inactivate the enzyme. cDNA was subjected to PCR using the Phusion Hot Start II DNA polymerase (reference F549S, Thermo Fisher) following the manufacturer’s instructions. Reactions were performed as follow: 30 s at 98 °C, 35 cycles comprising 10 s at 98 °C, 15 s at 64 °C, 20 s at 72 °C and final step at 72 °C for 5 min. Partial sequencing of each segment was performed in both directions with the BigDye terminator kit (ThermoFisher) on a 3130 sequence analysis (Applied Biosystems).
